# A Survey on Handover and Mobility Management in 5G HetNets: Current State, Challenges, and Future Directions

**DOI:** 10.3390/s23115081

**Published:** 2023-05-25

**Authors:** Yasir Ullah, Mardeni Bin Roslee, Sufian Mousa Mitani, Sajjad Ahmad Khan, Mohamad Huzaimy Jusoh

**Affiliations:** 1 Centre for Wireless Technology, Faculty of Engineering, Multimedia University, Cyberjaya 63100, Malaysia; 1221400715@student.mmu.edu.my; 2 Head of Next Generation Network Research Institute, Telekom Malaysia Research & Development, Cyberjaya 63000, Malaysia; sufian@tmrnd.com.my; 3 Department of Computer Engineering, Hoseo University, Asan-si 31499, Republic of Korea; dr.sajjadkhan19@gmail.com; 4 School of Electrical Engineering, College of Engineering, Unversiti Teknologi MARA, Shah Alam 40450, Malaysia; huzaimy@uitm.edu.my

**Keywords:** fifth generation (5G), handover (HO), heterogeneous networks (HetNets), mobility management (MM), quality of services (QoS), key performance indicators (KPIs)

## Abstract

Fifth-generation (5G) networks offer high-speed data transmission with low latency, increased base station volume, improved quality of service (QoS), and massive multiple-input–multiple-output (M-MIMO) channels compared to 4G long-term evolution (LTE) networks. However, the COVID-19 pandemic has disrupted the achievement of mobility and handover (HO) in 5G networks due to significant changes in intelligent devices and high-definition (HD) multimedia applications. Consequently, the current cellular network faces challenges in propagating high-capacity data with improved speed, QoS, latency, and efficient HO and mobility management. This comprehensive survey paper specifically focuses on HO and mobility management issues within 5G heterogeneous networks (HetNets). The paper thoroughly examines the existing literature and investigates key performance indicators (KPIs) and solutions for HO and mobility-related challenges while considering applied standards. Additionally, it evaluates the performance of current models in addressing HO and mobility management issues, taking into account factors such as energy efficiency, reliability, latency, and scalability. Finally, this paper identifies significant challenges associated with HO and mobility management in existing research models and provides detailed evaluations of their solutions along with recommendations for future research.

## 1. Introduction

The rapid growth in mobile user equipment (UE) and advanced applications has led to a significant rise in the demand for data traffic [1]. As a result, the telecommunication industry, the 3rd generation partnership project (3GPP), and the international telecommunication union (ITU) require assistance in upgrading their infrastructure to meet these demands. Modern tools such as the internet of things (IoT), device-to-device (D2D), vehicle-to-vehicle (V2V), and the COVID-19 pandemic have considerably increased the demand for content-of-interest services. Additionally, online services such as telemedicine, telemarketing, online conferences and meetings, and online shopping have necessitated the development of high-capacity, improved spectral efficiency (SE), and low-latency wireless communication systems (WCS) [2]. The 5G technology is regarded as the best solution to provide high coverage, higher data rates and capacity, extremely low latency, improved energy efficiency and SE, and support for a wide range of devices and services [3,4,5,6,7,8].

To provide better coverage, higher throughput, and excellent connectivity for both indoor and outdoor users, the 5G network utilizes enabler solutions such as network densification or heterogeneous network (HetNet) [9,10], millimeter wave (mm-Wave) [11,12], and massive multiple-input–multiple-outputs (M-MIMO) [13]. In 5G and beyond-5G (B5G) networks, quality of service (QoS) and quality of experience (QoE) are the important factors for improving handover (HO) and mobility performance to provide seamless and uninterrupted service to users. QoS guarantees that a network satisfies the service requirements of its users, while QoE measures how well the user perceives the service. Factors such as bandwidth, network coverage, received signal strength (RSS), latency, jitter, packet loss, HO performance, and UE capabilities impact QoS and QoE in 5G/B5G HetNet. The consideration of these both in the optimization of HO and mobility management processes can empower network operators to provide a superior and satisfactory user experience [14,15,16,17]. Since sub-6 GHz is no longer sufficient to accommodate the vast number of UEs and ensure the best QoS and QoE for IoT devices within a 5G system, a new frequency band, i.e., mm-Wave, needs to be utilized [11]. The mm-Wave operates at very high frequencies, ranging from 24 GHz to 84 GHz for 5G, and it may be increased up to 100 GHz in the future. Due to the wider bandwidth, mm-Wave can resolve the issue of capacity requirements in 5G. However, it significantly suffers from higher path loss and cannot penetrate walls or other obstacles due to its shorter wavelength. Increased frequencies can cause a severe issue, strengthening the HO problem [18].

Similarly, HetNet deploys different kinds and sizes of small cells such as micro, pico, and femtocells over the macro base station (MBS) to provide service to UEs. HetNet has the ability to enhance system capacity in terms of user density, improving the coverage area, and providing uninterrupted connectivity to users with higher data rates [9,10]. Massive MIMO is also considered a potential technique for the NG-5G cellular networks since it can dramatically increase SE [19,20]. Massive MIMO technology, in which the BS is equipped with a large number of transmitting and receiving antennas, can serve multiple users and entertain a maximum number of UEs simultaneously [13].

Seamless HO is one of the significant challenges in 5G ultra-dense HetNet (UD-HetNet) to control the mobility of high-speed users [21]. Efficient HO is required for mobility management in WCS to switch UE from one cell to another without any interruption or disconnection. However, in 5G HetNet, transferring UE data between cells or networks results in computational complexity and signaling overhead, leading to significant HO implications. The problem of mobility becomes more challenging with frequent HO (FHO) among small cells. The occurrence of FHO of UE not only increases network congestion and signaling overhead but also incurs additional costs associated with relocating services back and forth with every HO. Along with the benefits of HetNet and mm-Wave, the deployment of a large number of small cells (HetNet) and the use of a high-frequency band in 5G HetNet also encounter mobility and HO problems. For instance, the small coverage area of the small cells increases the likelihood of HO, HO failures (HOF), HO ping-pong (HOPP), and radio link failure (RLF), which adversely affect the capacity, throughput, and SE of the entire system.

**Figure 1:**
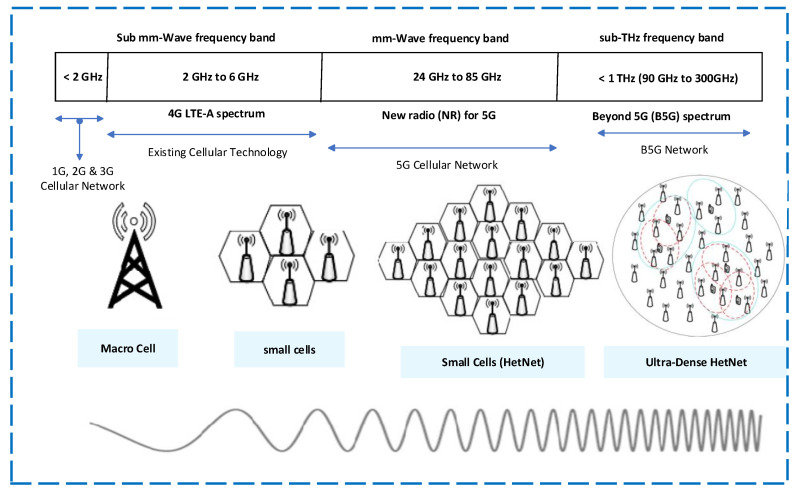
Frequency spectra and architectures of various cellular networks.

Figure 1 depicts the frequency spectrum and structural layout of various cellular networks. The implementation of UD-HetNet, where the number of cells in an area is considerably greater than the active number of users, is essential to meet 3GPP specifications in the 5G network [22,23,24]. However, the fundamental issue of ensuring a seamless, ongoing, and dependable connection for high-speed mobile users in the 5G UD-HetNet needs to be addressed [25]. According to Ericsson’s latest mobility report, the number of cellular subscribers, currently around 8.2 billion, will reach 9.1 billion by the end of 2027. In the ultra-high-speed 5G UD-HetNet scenario, HO management is crucial for enhancing the system’s overall performance in terms of throughput, resistance to unnecessary HO (UHO), and RLF. Extreme and FHO will lower the QoS and consume more scarce resources. Therefore, to ensure seamless and uninterrupted connectivity during high-speed scenarios, it is crucial to develop and implement new techniques for managing mobility and HO in 5G UD-HetNet networks.

### Motivation and Major Contributions

The UD-HetNet-based 5G technology satisfies the demands of the present era by providing faster data rates, lower latency, and higher user density. The higher cell density in UD-HetNet, with a large number of small cells operating at low power, ensures ubiquitous and high-capacity connectivity [26,27]. These low-power small-cell deployments improve capacity, coverage, and reliability and enhance user performance at the cell edge. However, the introduction of small cells in the 5G network also brings some drawbacks. Due to the limited frequency range and the vast number of small cells, frequency reuse in 5G leads to co-channel interference (CCI). Additionally, small cells utilize the mm-Wave frequency spectrum due to their short range and susceptibility to signal interference. Furthermore, high-speed UEs quickly cross multiple cells due to the small cells’ limited coverage area, leading to FHO, which results in HOF and HOPP effects. In the presence of FHO, the UE will consume additional energy to connect with the appropriate cells.

In HOPP, the UE temporarily connects to a nearby/target cell due to rapid fluctuations in RSS from both the serving and target cells and then bounces back to the original serving cell. Unnecessary HO significantly degrades the performance of future WCS, which also results in inefficient resource usage and excessive power consumption. User mobility is the primary driver of HO. The optimal time for HO can be determined using many HO decision parameters, including the UE’s location, the direction and pattern of the user’s mobility, the type of cell, the availability of radio resources, the user’s behavior and preferences, the battery level, the environment, and the data and traffic volume in a specific cell. The UE switches cells based on these attributes to deliver uninterrupted, secure, and timely services. To ensure seamless and reliable connectivity and minimize frequent and UHOs, the number of handovers (HOs) must be mitigated for 5G UD-HetNet. The main factors that disrupt seamless connectivity in UD-HetNet include mm-Wave, a massive increase in the number of connected UEs, massive deployment of small cells (HetNet), high-speed users, overlapping network deployment (small cells’ coverage area coinciding with macro cells’ coverage area), and the implementation of dual connectivity (DC), where UEs connect to two BSs simultaneously.

This study aims to compare alternative HO and mobility management strategies and algorithms in UD-HetNet for 5G cellular systems based on numerous key performance factors (KPIs). The article’s major contributions are as follows:

1. Thorough analysis of 5G HetNet’s setup adjustments concerning HO failures (HOFs) and mobility management issues;
2. Examination of the KPIs of current works, including HO and mobility issues, with the remedies and applied standards;
3. Evaluation of the current model’s performance against HOF and mobility management issues based on throughput, energy efficiency (EE), reliability, latency, and scalability;
4. Highlighting the significant challenges associated with HO and mobility management in the current research models in 5G HetNet and discussing potential recommendations for solutions.

**Figure 2:**
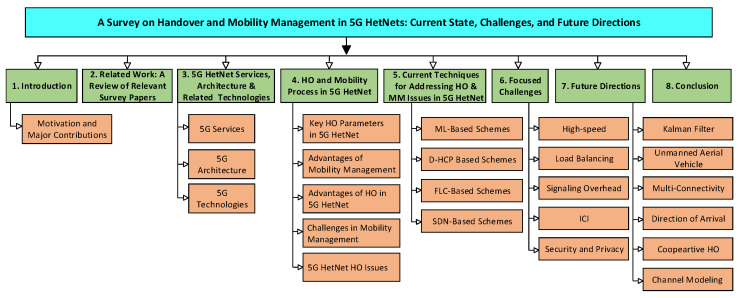
The outline of the paper.

The outline of the paper is given in Figure 2. The rest of the paper is organized as follows: Section 2 covers related work and presents an overview of relevant survey papers. Section 3 discusses 5G HetNet services, architecture, and related technologies. Section 4 explains the HO and mobility process in 5G HetNet, while Section 5 explores the current technologies for addressing HO and MM issues in 5G HetNet. Section 6 focuses on the challenges related to HO and MM, while Section 7 discusses future directions. Finally, the paper is concluded and summarized in Section 8.

## 2. Related Work: A Review of Relevant Survey Papers

A number of surveys have been published on HO and mobility management issues in 5G HetNet with applied techniques to mitigate them, which are discussed with limitations as follows. The main concept of HO and mobility management and how to improve their performance through the dual connectivity (DC) approach, where a UE can simultaneously connect to two different radio access technologies (RATs), is reviewed in [28] for future UD-HetNet. Various HO and mobility management techniques, such as machine learning (ML)- and software-defined networking (SDN)-based schemes, are discussed to enhance mobility issues. The key aspects of future HO probabilities over DC in existing and NG-WCS are highlighted. Furthermore, the authors also focused on several HO/mobility management challenges, such as high-speed scenarios and security and privacy issues in UD-HetNet, which highly degrade the performance of the WCS. However, load balancing (LB) and inter-cell interference (ICI) have not been considered during the HO process, and they can severely affect mobility performance and increase the HOF rate in the UD-HetNet environment. Furthermore, the article does not comprehensively review all HO management techniques proposed for 5G networks, further limiting its scope.

The basics of mobility management in 5G UD-HetNet, mobility management requirements, architecture, and issues related to mobility management have been extensively reviewed in [29]. Radio resource control (RRC) inactive status, initial access, registration, and the paging procedure have been discussed in detail. The authors have also examined different kinds of mobility—cell level and beam level mobility—used in 5G UD-HetNet to provide seamless and uninterrupted connectivity. In addition, the survey paper has covered modern methods, such as ML, SDN, and fuzzy-logic controller (FLC), that are utilized for estimating HO and mobility management issues. Further, challenges such as load balancing, signaling overhead, and security and privacy issues followed by effective solutions to overcome these issues in 5G UD-HetNet have been addressed and demonstrated. Although this paper exhaustively reviewed the various aspects of 5G UD-HetNet, it still lacks high-speed scenarios and ICI challenges. High speeds can cause HOF due to FHO, and ICI is produced by the presence of multiple-RATs (Multi-RATs) in HetNet, which can disrupt the HO and mobility performance.

In [30], a concise and clear overview of the advancements in mobility management within the evolving 5G architecture has been provided. It explores the current approaches to vertical HO (VHO) in 5G HetNet, considering the impact of new architectural elements such as SDN, NFV, and mobile edge computing (MEC) while maintaining media-independent HO procedures. Moreover, the authors analyzed the requirements of the three main service types and the related proposals for distributed mobility management within 5G HetNet. In addition, the evolutionary steps in mobility management signaling, aligning the QoS requirements for various 5G service scenarios, and vertical use cases have been highlighted. Nevertheless, the authors have only explained the SDN- and D-HCP-based HO management schemes and have yet to address the challenges encountered by UE during the HO process in a UD-HetNet.

The emergence of innovative mobile networks, such as IoT, machine-to-machine (M2M), D2D, and vehicle-to-everything (V2X), exacerbates mobility issues, which significantly deteriorates HO performance in terms of HOF, HOPP, and RLF in future UD-HetNet. In reference [31], the authors examine the general concept of mobility management and the key factors that cause HO and mobility issues in 5G HetNet. The work also discusses the existing studies on mobility management and its characterizations in the 5G HetNet, particularly exploring ML- and D-HCP-based approaches. Furthermore, the study highlights the key issues that UEs face throughout the HO/mobility process and future research directions on mobility management in 5G HetNet and B5G. Despite a thorough discussion on mobility and HO management techniques, the article fails to address critical factors such as high-speed scenarios, ICI, and security and privacy, which significantly affect the performance of HO and mobility in 5G HetNet.

In reference [32], the authors discuss the background of 5G, services, different models of 5G architecture, 5G benefits, and their impacts on modern applications. The article also studies 5G key technologies that enhance QoS and meet next-generation WCS (NG-WCS) requirements. Moreover, the authors examine various challenges encountered by UEs in the 5G UD-HetNet environment and existing ML-based solutions applied to overcome 5G challenges. The authors in [32] also discuss ML- and SDN-based schemes to further improve HO and mobility performance in 5G UD-HetNet. In addition, a few HO/mobility management-related issues such as ICI, security, and privacy have been taken into account to enhance HO performance in terms of various KPIs in the 5G system. However, HO in 4G/LTE-Advance systems and other key challenges that significantly degrade the HO and mobility performance of 5G UD-HetNet still need to be addressed.

A survey on HO and mobility management in 5G ultra-dense small cell (UDSC) networks using ML-based algorithms is presented in [33]. The article comparatively analyzes various ML- and fuzzy-logic controller (FLC)-based HO and mobility management schemes. The future research directions and challenges for 5G-UDSC networks are also briefly addressed in the paper. Nevertheless, the omission of a discussion on the issue of frequent and UHOs caused by high-speed users in UDSC environments is a limitation of this article.

In [34], the authors address HO and mobility management issues in 4G-LTE HetNet using ML and SDN algorithms. The article discusses challenges such as load balancing and ICI that impact HO performance in 4G/LTE. However, a major drawback of this article is that it fails to address the HO/mobility management issue in 5G UD-HetNet, which should also be considered.

Article [35] provides an overview of HO and mobility management in future mobile networks, with a focus on optimizing mobility robustness. It covers challenges in managing mobility in HetNet and techniques to enhance mobility management. The potential of emerging technologies such as ML, AI, blockchain, and edge computing is also mentioned. However, its relevance may be limited to those interested in emerging technologies as it lacks practical insights for real-world networks and a wider social and economic context.

In [36], the authors comprehensively examined mobility and HO management in LTE and 5G-NR with UD-HetNet wireless networks. They explored various aspects of HO management, including performance metrics, HO types, types of HOF, differences between LTE and NR mobility procedures, and potential mobility enhancers. The paper also discussed research challenges and techniques related to HO and mobility management in 5G-NR, including high-speed scenarios, beam management, and beam mobility. The authors explored ML- and FLC-based techniques as possible solutions to overcome HO and mobility management issues in 5G-NR. However, the paper noted that the analysis did not consider some important HO performance challenges, such as ICI, load balancing, and signaling overhead. The authors in [37] present a comprehensive review of 5G-enabling technologies with UD-HetNet. They also address research challenges and opportunities related to intelligent or ML-based HO and mobility management techniques and backhaul solutions for 5G wireless networks. These challenges include issues such as unnecessary HOs, imbalances in radio resource sharing, high energy consumption, intense ICI, and reduced QoS. However, the authors did not consider user speed, which is a major issue for HO and mobility performance improvement in future UD-HetNet.

In addition to these survey articles, there are some other articles that provide extensive reviews of HO and mobility management in HetNet and UD-HetNet. The authors in [38] comprehensively review the challenges and solutions related to mobility management in advanced communication networks such as 5G and B5G. They cover various existing HO and mobility management schemes, including traditional and advanced approaches such as SDN and NFV. Furthermore, future challenges such as managing a large number of devices, ensuring seamless handovers, addressing security and privacy concerns, and improving resource utilization are also discussed. However, the paper does not specifically address challenges related to high speed, load balancing, and ICI, and focuses primarily on SDN and NFV as the advanced mobility management schemes. In [39], the authors thoroughly reviewed the HO mechanisms in mobile HetNets, with a focus on providing a detailed and latest analysis of the current state of HO management. The authors discuss the basic procedures for HOs, the impact of HCPs on KPIs, self-optimization techniques, and the major challenges faced in HO management. Additionally, the authors categorized existing HO decision algorithms, such as ML/DL, RSRP, FLC, and velocity-assisted HO techniques, and reviewed evaluation methods for performance. A survey of SDN-enabled architectures and HO optimization techniques in multi-RAT networks is conducted in [40]. The advanced SDN-enabled architectures for radio access networks (RANs), offloading techniques, and implementation strategies are investigated in this paper. Moreover, the authors discuss the challenges and limitations of SDN-based HO optimization approaches and suggest potential areas for future research. The article [41] provides a state-of-the-art survey of MRO studies that utilize ML techniques, addressing the methodology used, HO criteria, HCPs, KPIs, simulators, and accomplishments. The study also discusses MRO challenges for intra-system and inter-system mobility and highlights issues for future investigations.

A brief description of survey articles from [28,29,30,31,32,33,34,35,36,37] is shown in Table 1, whereas Table 2 summarizes the scenarios, applied schemes, and focused challenges discussed in the related surveys.

## 3. 5G HetNet Services, Architecture, and Related Technologies

This section discusses 5G services, the basic architecture of 4G LTE-Advanced (LTE-A) and 5G-NR, and key technologies that enhance network capacity, reliability, and connectivity.

### 3.1. 5G Services

The three primary 5G-NR services are Enhanced Mobile Broadband (eMBB), Massive Machine-Type Communications (mMTC), and Ultra-Reliable Low-Latency Communications (URLLC). These services aim to utilize 5G technologies to meet the requirements of various 5G applications.

- **Enhanced Mobile Broadband:** The anticipated benefits of 5G systems over LTE-A systems include increased data rates (20 Gbps in DL and 10 Gbps in UL) and huge capacity (10^6^ devices/km^2^) [42]. As a result, applications such as streaming HD video, online gaming, and other high-bandwidth services are expected to perform better on the 5G network [43]. Two significant technologies, mm-Wave and M-MIMO, are used to deliver high data rates in 5G networks [44].
- **Massive Machine-Type Communications:** Massive machine-type communication (mMTC) provides scalable connectivity solutions for a massive number of devices and supports a large number of devices using MEC as a technology solution [45]. The mMTC 5G service is well-suited for applications such as the IoT and M2M communications [46].
- **Ultra-Reliable Low-Latency Communications (URLLC):** URLLC 5G service is based on NFV, SDN, and MEC technologies. URLLC is expected to be the most challenging service to provide, requiring a high level of network availability and low latency. The 5G network ensures ultra-reliable communication with a low packet-error rate (<$10^{-5}$). Furthermore, for services such as industrial automation, autonomous vehicles, and remote medical procedures (healthcare), URLLC is a promising solution [30,47].

### 3.2. 5G Architecture

Compared to 4G LTE, 4G LTE-A is designed to enhance capacity, increase data rates, and improve system efficiency. These improvements are achieved using advanced technologies and features such as carrier aggregation (CA), MIMO, and coordinated multiple points (CoMP). CA allows multiple frequency bands to be used simultaneously to increase data rates, while MIMO enhances signal quality and capacity. In CoMP, a multiple-evolved Node B (eNB) transmits signals to a UE, enhancing Inter-Cell Interference Coordination (eICIC) to improve network capacity by reducing interference between cells.

The 4G LTE-A architecture comprises numerous key elements, including UE, eNB (BS), core network (CN), and Evolved Packet Core (EPC), among others. LTE-A relies on interconnected eNBs (BSs), where a UE establishes a connection with the network via an eNB. The eNBs provide coverage to all UEs within their coverage area and are connected to the core network through a gateway. The UEs and eNBs are linked via the Uu interface, while the eNBs are connected through an IP-based logical X2 interface. The S1 interface links all eNBs to the core network (CN) of LTE-A, which provides the control and management functions of the network. The CN, also known as the evolved packet core (EPC), is the pillar of LTE-A architecture that connects the eNB to other elements of the CN and provides necessary functions for advanced features such as CA and inter-cell interference coordination (ICIC). The 4G/LTE-A operates on sub-6 GHz frequency bands and supports a wider bandwidth of 100 MHz, which is five times higher than the previous version of cellular technology (3G/LTE). The most commonly used frequency bands for 4G/LTE-A are 700 MHz, 900 MHz, 1800 MHz, 2100 MHz, and 2600 MHz.

The 5G architecture includes the same components as 4G/LTE-A, but the eNB in LTE-A is replaced with gNB. The 5G radio access network (RAN) is based on NR technology, which delivers faster data rates, lower latency, and larger capacity than existing cellular technologies. The 5G-NR utilizes both the sub-6 GHz spectrum (non-mmWave) and mmWave band (high frequency). The former is used to provide service over a wider coverage area due to better penetration depth and lower penetration losses, while the latter is responsible for serving users over a smaller coverage area due to high penetration losses [48,49,50]. In addition, 5G is capable of supporting wider bandwidths up to 400 MHz, which represents a substantial increase compared to 4G LTE-A. In addition to NR, 5G uses several other key technologies such as Massive-MIMO, HetNet, beamforming, network slicing, SDN, and NFV for enhancing system capacity, increasing throughput, reducing interference, improving QoS, and enabling the network to be easily reconfigured to meet changing requirements, respectively.

**Figure 3:**
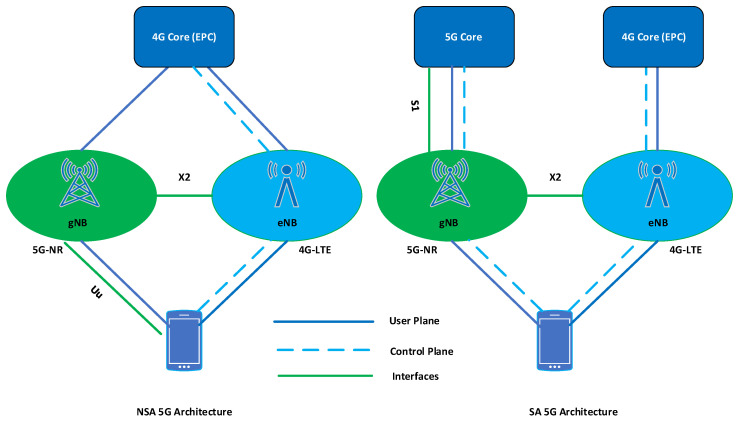
The 5G SA and NSA architecture.

Standalone (SA) and non-standalone (NSA) are two different deployment models in 5G systems. NSA-5G architecture depends on LTE-A core (EPC). In the NSA model, the existing 4G/LTE-A infrastructure and technology (core network) is responsible for the control plane, and 5G-NR technology is only utilized for the data plane. On the contrary, the SA-5G network does not depend on the LTE-A core network (CN). In SA-5G architecture, both the data plane and control plane are served by the 5G core (5GC). Compared to NSA-5G, the SA-5G architecture provides better performance and capabilities in terms of data rate, capacity, and throughput [51]. Figure 3 illustrates the basic architecture, components, and interfaces of 4G LTE-A and 5G Networks (SA and NSA).

### 3.3. 5G Technologies

The most important enabler technologies for 5G cellular systems are HetNet, mm-Wave, Massive-MIMO, beamforming, full-duplex (FD), and D2D technologies. These technologies fulfill the requirements of future networks and have achieved high capacity, enhanced data rates, and massive and seamless connectivity of UEs.

#### 3.3.1. Heterogeneous Network

Heterogeneous networks (HetNets) are a promising technology for 5G and Beyond 5G (B5G) that offers high capacity, high data rates, low latency, and wider coverage, especially to UEs at the cell edges. HetNet utilizes a mixture of MBSs underlaid with small base stations (SBSs) or small cells (SCs) and different RATs to provide cellular service and fulfill the demand of NG-WCS. The MBSs and SBSs operate dynamically over different spatial and time scales. To ensure full coverage and achieve high data rates in densely populated environments with tall buildings and obstacles, the deployment of SCs plays a critical role. The basic idea behind SC deployment is to bring the network closer to users. The SCs are generally categorized as femtocells, picocells, and microcells, where femtocells are the smallest and can handle a few users (5–10 users), picocells are in the middle, covering 250 m and can handle 100 users, while microcells are the largest, covering 1–2 km and can be used in large events [52].

Due to their low power consumption, low cost, easy installation, and reliability features, SCs have been incorporated into the 5G network. The SCs can be installed both inside and outside of buildings, providing excellent service to subscribers. Low cost refers to the cost of deploying a single 5G cell rather than the deployment cost of the entire 5G system. The deployment of SCs also reduces the burden on macro cells and improves the UEs’ QoS by utilizing a cell load-balancing strategy [53]. The installation of UD-HetNet over macro-cells is beneficial for both operators and users and effectively maximizes the capacity of the 5G system [54]. The main effect of UD-SC deployment is the ICI between the macro cell (MC) and the SC, and this is further boosted when a UE within the coverage of the SC receives signals from the MC. As a result, the SINR is reduced in UD-SC, which affects the network performance and user experience [55,56]. In addition, the performance of the network is based on the number of successful HOs during user mobility. Users may be high-speed (such as those in fast vehicles) or very low-speed (such as pedestrians). Therefore, when planning the HO process, both of these scenarios should be taken into consideration to ensure the successful implementation of HOs in HetNet.

#### 3.3.2. Millimeter Wave

The millimeter-Wave (mm-Wave) band, also known as the extremely-high-frequency (EHF) band, is extensively used in 5G communication systems and operates within a frequency range of 30–300 GHz. The high-frequency band provides a large bandwidth of up to 800 MHz, which accommodates maximum channels and enhances the cell capacity in terms of users (high data rate and low latency) in 5G [57]. Due to the wider bandwidth, this technology (mm-Wave) ensures a higher bit rate, capacity, and improved network efficiency compared to existing generations, making it suitable for 5G communication systems [58]. Despite these advantages, mm-Wave has some limitations, such as high sensitivity to blockage and high path losses (due to buildings, trees, and other obstacles), which can severely degrade the performance of the 5G system. Because of its higher frequency, mm-Wave cannot travel fast and is unable to provide service to UEs at the cell edge or away from the BS. Thus, mm-Wave is used in HetNet due to its small coverage radius.

#### 3.3.3. Massive MIMO

The massive MIMO (M-MIMO) system equips a base station (BS) with hundreds or even thousands of transmitting and receiving antennas, allowing it to serve a large number of UEs simultaneously. The M-MIMO technique is necessary in the 5G network to handle the massive increase in connected UEs without congestion. Compared to the MIMO technology used in 4G systems, the deployment of M-MIMO in 5G HetNets can improve signal quality (QoS) and UE performance at the cell edge, enhancing cell throughput [59]. M-MIMO’s use of a large number of receiving and transmitting antennas also improves the data rate and signal-to-interference-plus-noise ratio (SINR) quality [60]. This technology can enhance the next generation of 5G by improving energy efficiency by up to 100 times and increasing capacity by up to 10 times.

Moreover, M-MIMO minimizes latency in the air interface and simplifies transceiver design [61,62]. Additionally, M-MIMO is a scalable technology that ensures uniformly excellent service to end-users. The integration of M-MIMO technology with UD-HetNets can be utilized to reduce issues of inter-cell interference (ICI) and load balancing in 5G HetNets.

#### 3.3.4. Beamforming

In WCS, beamforming is a technique that directs the transmission of a signal in a specific direction instead of spreading it equally in all directions. Beamforming is an essential component of 5G technology, enabling 5G networks to deliver high-speed, low-latency, and reliable communication services. It is crucial to realize the full potential of 5G by enhancing the coverage, capacity, and quality of the wireless signal. The beamforming technique allows 5G networks to focus the energy of the signal toward the user device, providing a more robust and stable connection. The beamforming technology improves the signal-to-noise ratio (SNR). It reduces the impact of interference, which is especially important in dense urban areas where multiple devices are trying to access the network. One of the benefits of beamforming in 5G is its ability to dynamically adjust the beam direction in real-time to adapt to changing environments, such as user device mobility. This feature helps to ensure a reliable and steady connection, even when the user device is on the move [63,64,65,66,67].

There are two main types of beamforming utilized in 5G UD-HetNet: analog beamforming and digital beamforming. Due to the use of multi-RATs, the 5G system utilizes both analog and digital beamforming approaches to enhance the system’s throughput and reliability.

In addition, analog beamforming uses amplifiers and phase shifters to adjust the signal phase and amplitude at each antenna element to achieve the desired shape of the beam. This approach is well-suited for mm-Wave frequencies, where high-gain antennas are required to compensate for the high signal loss. Digital beamforming, on the other hand, uses mathematical algorithms to process the signal in the baseband processing unit. This approach provides flexibility in the frequency spectrum, as the beam direction can be changed dynamically based on the changing environment. Digital beamforming is well-suited for sub-6 GHz frequencies, where the frequency spectrum gain is lower but more processing power is available [68].

#### 3.3.5. Full-Duplex Communication in 5G HetNet

Full-Duplex (FD) communication is a key aspect of 5G HetNet that allows for the simultaneous transmission and reception of data on the same frequency band, delivering a range of benefits that are crucial to achieving in 5G [69]. One of the key advantages of FD communication is increased network capacity, as it eliminates the need for separate frequency bands for transmission and reception. This results in efficient use of the available spectrum and improved SE, leading to higher data rates and better network performance [70]. Additionally, FD communication provides real-time bidirectional communication, which is critical for supporting new 5G use cases such as augmented reality, virtual reality, and IoT applications. Furthermore, FD communication reduces latency, making it an ideal solution for applications that require low-latency communication. It also eliminates time-division multiplexing, which can result in increased reliability and reduced errors in the transmission of data [70,71].

FD communication also offers more flexibility and dynamic network configurations, enabling the network to adapt to changing traffic patterns and support new use cases. The bidirectional nature of FD communication also provides an additional layer of security, verifying the identity of the communicating parties in real-time. Interference is reduced as FD communication eliminates the need for frequency division, improving overall network performance. The use of a single frequency band also reduces the need for multiple frequency bands, resulting in cost savings associated with acquiring and managing spectrum licenses. Ultimately, FD communication delivers higher data rates, lower latency, and improved reliability, resulting in an improved user experience for subscribers and better support for new 5G use cases. In conclusion, FD communication is an essential component of 5G HetNet and will play a critical role in shaping the future of wireless communication.

#### 3.3.6. Device-to-Device Communication in 5G HetNet

Device-to-Device (D2D) communication in 5G HetNet refers to the direct communication between two devices without the involvement of a centralized network infrastructure (i.e., base station). D2D communication in 5G HetNet offers several benefits, making it an attractive technology for various applications. Firstly, D2D communication reduces the burden on the cellular network, leading to improved network capacity and reduced network congestion [72]. This means that more devices can be served simultaneously, leading to higher network utilization and a better user experience. Secondly, D2D communication enables direct communication between devices, leading to reduced latency and increased communication speed. This makes D2D ideal for real-time applications such as gaming, video streaming, and augmented reality. Thirdly, D2D communication provides improved coverage and connectivity in areas with weak or no cellular signals. This is particularly beneficial in remote areas where cellular coverage is limited. Fourthly, D2D communication is energy efficient, making it ideal for IoT devices that have limited power resources. Finally, D2D communication enables personalized and customized communication services, such as social networking and multimedia sharing, which enhance the overall user experience [73,74,75].

#### 3.3.7. Software-Defined Networking/Network Function Virtualization in 5G HetNet

The use of software-defined networking (SDN) and network function virtualization (NFV) in 5G HetNet offers several key advantages that help service providers meet the demands of 5G and provide high-quality services to their customers. Firstly, SDN allows for centralized network management and programmatic control, enabling faster and more efficient deployment of 5G services. Additionally, NFV enables the virtualization of network functions, which allows for the dynamic allocation of network resources based on real-time demand, resulting in improved network performance and reduced costs. Furthermore, SDN and NFV provide greater scalability and flexibility, enabling service providers to respond to changing demands and easily add, modify, or remove network functions as needed. Additionally, SDN provides increased visibility into network activity and enables centralized security management. In contrast, NFV enables the virtualization of security functions such as firewalls and intrusion detection systems, improving network security. Finally, SDN and NFV can be used to manage the delivery of different types of 5G services, ensuring that each service receives the necessary network resources to meet its specific requirements, thereby improving the QoS. Overall, the use of SDN and NFV in 5G HetNet provides service providers with increased automation, resource optimization, scalability, security, QoS, flexibility, and innovation [76,77].

**Figure 4:**
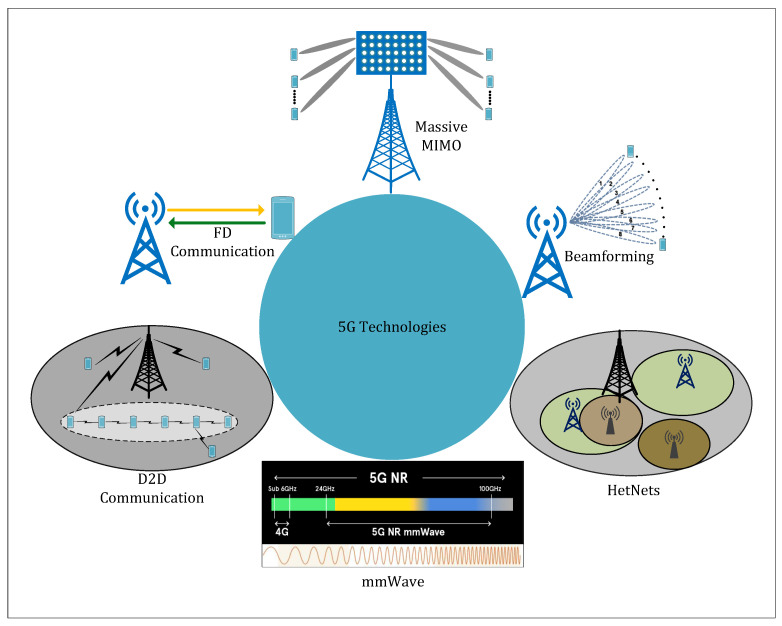
The 5G key technologies.

The deployment of various 5G key technologies for enhancing performance and fulfilling the demand of future wireless communication systems are discussed in several works [52,53,54,55,56,57,58,59,60,61,62,63,65,66,68,69,70,71,72,73,74,76,77,78] and are shown in Figure 4. The integration of UD-HetNet with 5G key technologies and their respective achievements are presented in Table 3.

## 4. Handover and Mobility Process in 5G HetNet

Handover (HO) refers to the process of a UE switching from the coverage of one cell to another without experiencing any interruption. The smooth and timely HO in any communication system is important for seamless connectivity. In WCS, HO is mainly categorized into Hard HO (H-HO) and Soft HO (S-HO), which are also known as break-before-make (BBM) and make-before-break (MBB) HO, respectively. In the H-HO process, the UE first terminates its connection from the old cell (serving cell) and then connects to the new cell (target cell) without any delays. In contrast, the S-HO process involves establishing a new connection to the next cell before terminating the existing connection [59].

Furthermore, Horizontal HO (HHO) and Vertical HO (VHO) are other types of HO. In the HHO process, the HO is performed within the same network, i.e., switching of UE from one cell to another using the same frequency [60]. As for VHO, it enables seamless UE mobility between different networks [61]. In other words, HHO occurs between homogeneous BSs, while VHO happens between HetNet BSs [79,80]. The HO process in 5G HetNet is performed based on VHO because of the overlapping deployment scenarios of different RATs (mm-Wave and Sub-6 GHz).

LTE-A utilizes the BBM-HO/H-HO technique to manage UE mobility between cells, which can result in increased HO interruption time (HOIT), data packet losses, and delay. The future WCS (5G and B5G) is based on HetNet and Ultra Dense HetNet deployment to enhance system capacity and provide ubiquitous connectivity.

In the HetNet environment, the HO process is expected to be faster and more redundant due to the smaller cell radius. The main feature of the utilization of VHO and S-HO techniques is to minimize HOIT, HO delay, and packet losses, which are important factors in 5G and B5G wireless networks.

The HO process in 5G new radio (5G-NR) is the same as that of 4G (LTE-A) HO and is composed of four phases: measurement (MR), HO initiation, HO execution, and HO termination or completion [81].

The HO process in 5G-NR involves several steps that enable UEs to switch from the coverage of one cell to another without interruption. The following is a list of steps that describe this process:

1. The UE receives signals from both the source gNB (s-gNB) and the target gNB (T-gNB) on the downlink, where the s-gNB is the serving cell and the T-gNB is the neighbor cell.
2. The UE measures the signal strength of these signals and creates a measurement report (MR).
3. The UE sends the MR to the s-gNB, and if the signal strength of the T-gNB is better than the s-gNB by a certain threshold during a predetermined time period, an HO event is initiated.
4. Based on the MR, the s-gNB sends an HO request (HO-req) to the T-gNB to initiate the HO process.
5. The T-gNB responds with an HO request Ack-command to indicate the availability of resources for HO execution.
6. After receiving the Ack-command, the s-gNB stops downlink data transmission and sends the HO command (HO-cmd) to the UE to initiate the HO execution process to the T-gNB.
7. In the execution phase, the UE disconnects from the s-gNB and establishes a connection with the T-gNB to synchronize.
8. Finally, in the HO completion phase, the T-gNB sends an HO complete command to the s-gNB to release the resources, and the downlink path is changed from the s-gNB to the T-gNB.

**Figure 5:**
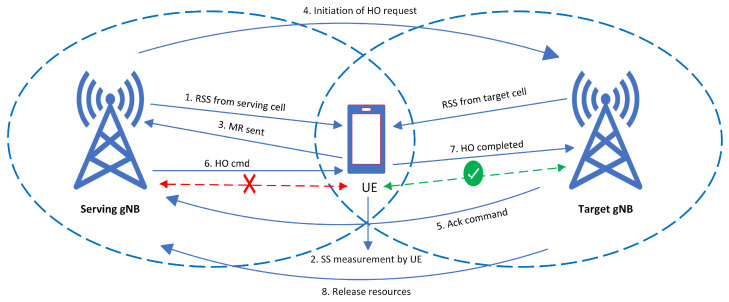
The 5G HO process.

The procedure for HO from initiation to completion is illustrated in Figure 5. The HO is typically initiated when signal quality from the serving gNB (S-gNB) is poor and interference from the target gNB (T-gNB) is very high. During the HO initiation phase, the UE may experience high RLF due to FHO, which is the most vulnerable period in the baseline HO process. The HO may fail if the HO control messages fail to be delivered or if RLF occurs during the HO procedure [82,83].

There are several conditions for triggering HO events, including A1, A2, A3, A4, and A5, but the most commonly used HO event in 5G is A3, where the T-gNB’s received signal strength (RSS) is higher than the S-gNB’s by a specified offset value. Table 4 provides a description of the various HO events and their triggering conditions.

**Figure 6:**
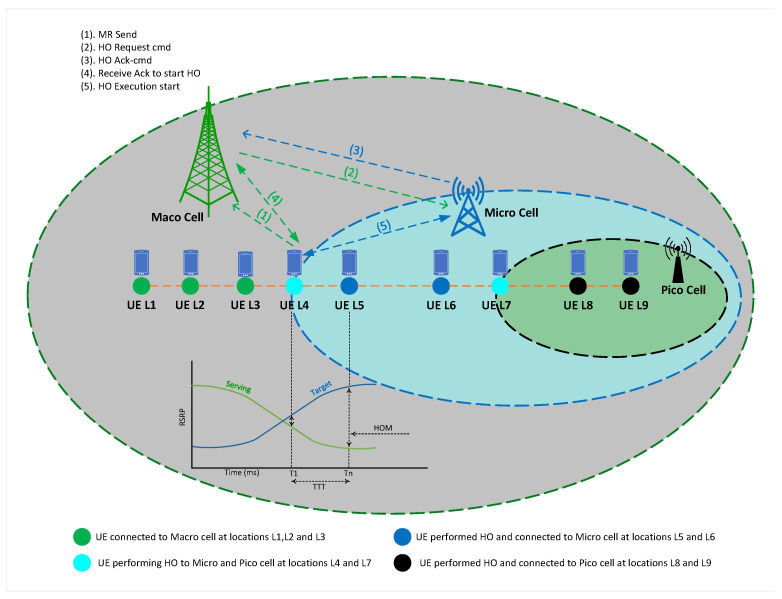
HO and mobility process in three-tier 5G HetNet.

The three-tier 5G HetNet’s HO and mobility process is illustrated in Figure 6. This network consists of macro, micro, and picocells, which are deployed to provide coverage and manage UE mobility. UEs located at L1, L2, and L3 are connected to the macrocell. However, due to strong RSS from other cells, UEs at L4 and L7 perform HO from the macrocell to the microcell and from the microcell to the picocell, respectively. L5 and L6 are locations where UEs are connected to the microcell, while L8 and L9 are locations where UEs are connected to the picocell.

### 4.1. Key HO Parameters in 5G HetNet

There are three important parameters considered for the HO procedure in 5G HetNet: HO decision parameters (HODPs), HO control parameters (HCPs), and key performance indicators (KPIs). These parameters play a crucial role in managing user mobility and enhancing HO performance in 5G HetNet and are discussed below.

#### 4.1.1. HO Decision Parameters

HO decision is a critical step in the HO initialization process in a communication system. Making perfect and appropriate HO decisions can enhance the performance of 5G HetNet in terms of reliability and seamless connectivity. In 5G HetNet, the HO decision is based on various parameters such as reference signal received power (RSRP), reference signal received quality (RSRQ), received signal strength indicator (RSSI), UE location, UE speed and direction (mobility pattern), cell load, SINR value, radio resources availability, UE battery level, RAT, HO priority (user behavior), and channel state information (CSI).

These parameters are crucial in making the HO decision process more efficient, effective, optimal, and secure. RSRP measures the power level of the reference signal received by the UE without noise and interference. It is used to specify the quality of the connection between the UE and BS and is usually measured in dBm. A higher RSRP value indicates that the received signal quality is strong and vice versa. RSSI measures the cellular signal strength, i.e., the total received power, including noise and interference, while RSRQ is the ratio between RSRP and RSSI, which is used to measure the quality of the reference signal received by the UE. These three HODPs are mostly used together to detect the best cell for HO in 5G HetNet environments.

The location of the UE can also be used to determine the best target cell for HO based on factors such as signal strength (RSSI) and coverage. The mobility patterns of the users, such as their speed and direction of travel, can be used to predict future HO requirements and optimize the HO process accordingly. If the UE is moving toward the target cell at high speed, its connection with the source cell becomes weaker while its connection with the target cell becomes stronger, and frequent HO will be performed. Based on the cell load or traffic, the UE from the heavily loaded cell can be switched to a less-loaded neighbor cell (load balancing) to perform secure and timely HO.

SINR of the UE is also essential to consider in the HO process. A UE, particularly at the cell edge, has low SINR because of the large distance from the BS, and the high interference value will cause HO to the appropriate neighbor cell. The availability of resources, such as bandwidth or processing power, at the current and target cells can be used to determine the optimal time for the HO in HetNet.

The battery level of a UE is an important parameter that can be used to determine whether the device has sufficient power to complete the HO process. In a 5G HetNet environment, which aims to provide services to MC and SCs UEs, the deployment of different RATs requires consideration of the serving and target cell’s RAT as a parameter in the HO decision process for optimal target-cell selection.

The behavior of the users, such as the types of applications they are using or the amount of data they are transmitting, can also be used for HO decisions. For example, if users are streaming HD video, they may require a high-quality connection with low latency, and the network may need to prioritize their HO requests accordingly. Another important parameter for making HO decisions is CSI, which estimates the condition of the radio link (attenuation, path loss, capacity, and interference) between the UE and the BS to select the best cell in the neighbor.

#### 4.1.2. HO Control Parameters

The HO Control Parameters (HCPs) are essential in 5G HetNet for optimizing and controlling the HO process to maintain network connection quality between the serving and target cell. HCPs control when and how a UE needs to HO to ensure connectivity to the best available cell at all times. In 5G HetNet, HO margin (HOM), time-to-trigger (TTT), and Cell Individual Offset (CIO) are commonly used HCPs that significantly contribute to maintaining the connection quality of UEs.

HOM is measured in dB and represents the difference in RSS or quality between the serving cell and the target cell. In high-speed scenarios, a lower HOM is preferred to prevent too-late HO (TLHO), while in low-speed environments, a higher HOM is suitable to avoid too-early HO (TEHO). In 5G HetNet, the HOM is an important parameter that directly affects HO performance. It should be set lower than in previous generations to achieve the high-speed and low-latency requirements of 5G. Additionally, the HOM should be optimized to balance the HO rate, HOF rate, and HO interruption time.

TTT is the time that a UE needs to spend below the trigger threshold before a HO is triggered. In 5G HetNet, TTT affects HO performance and overall QoS. A lower TTT can improve the HO rate and user experience but increase HOF rate, while a longer TTT can decrease the HO rate but reduce the QoS and user experience. The value of TTT should be carefully set based on the specific use case and network requirements.

The CIO is used to measure the signal level of a neighboring cell relative to the serving cell and adjust the HO decision criteria accordingly. In 5G HetNets, where multiple cells of varying sizes and technologies coexist, it is crucial to ensure that the UE is handed over to the best available cell based on the network conditions and the user’s location. The value of the CIO is typically set during network design and is based on network requirements.

To significantly improve the HO process, optimizing only one HCP is insufficient, and all HCPs need to be optimized according to UE requirements to enhance the HO KPIs. To reduce HOF and HOPP rates in 5G HetNets, the values of HCPs, including HOM and TTT, should be low for high-speed UEs and high for UEs moving at a low speed. In HetNets, due to their small coverage area and high-speed UEs, the HCPs are set to be as low as possible to improve HO performance in terms of HOF and HOPP and to achieve high capacity in terms of UE connectivity and reliability.

Mobility robustness optimization (MRO) and load-balancing optimization (LBO) are key schemes for enhancing HO performance by adjusting HCP values and offloading users to the best cell in the neighborhood, respectively, where they can attain excellent QoS. The primary goal of MRO is to avoid too-early HO (TLHO), too-late HO (TEHO), and unwanted or wrong HOs, while LBO significantly improves UE throughput [84]. In LBO, the UE performs an early HO to cells in the surrounding area that have less traffic load, which provides sufficient resources by adjusting HCP values [85,86,87].

**Figure 7:**
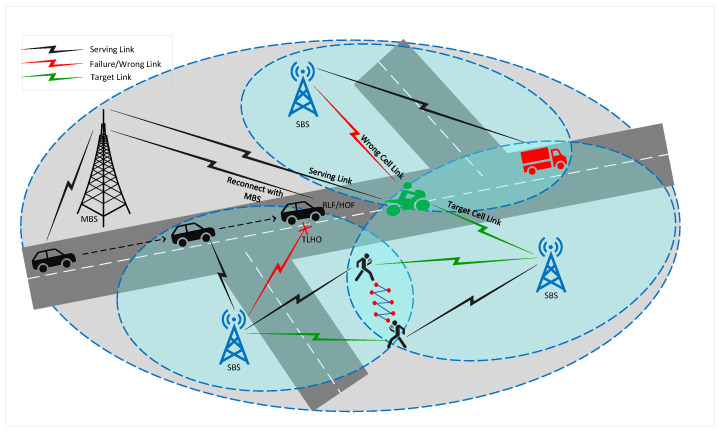
The 5G HetNet with different mobility users and their related issues.

In a 5G HetNet, different types of mobile users, such as pedestrians, vehicular users, and high-speed users, pose unique challenges related to mobility and connectivity. Pedestrian users may experience HOPP due to their slower speed, while high-speed users may experience a higher rate of RLF due to their rapid movement and the limited time available for the network to establish and maintain connections. A wrong cell HO in HetNets can be caused by various factors, including interference from neighboring cells, inadequate signal strength, and network congestion. Figure 7 depicts the 5G HetNet with different types of mobile users and their related issues.

#### 4.1.3. Key Performance Indicators for Handovers

In a HetNet, Key Performance Indicators (KPIs) refer to a set of measurements used to evaluate network performance related to HO and mobility. These KPIs include HO rate (HOR), HO failure rate (HOFR), HO success rate (HOSR), HO delay (HOD), HOPP, RLF rate, HO execution time (HOET), HO interruption time (HOIT), packet loss, energy consumption, and signaling overhead due to unnecessary FHO. These KPIs are critical for assessing how well the network meets the needs of its users and for identifying areas for improvement. These KPIs also offer many benefits, including enhancing network efficiency, reliability, and user experience. Besides their advantages, these KPIs also have potential limitations that should be taken into consideration. These limitations include a lack of standardization in defining and measuring KPIs, inadequate reflection of network performance or end-user experience, suboptimal selection of KPIs, and incomplete assessment of network performance. To improve 5G performance, particularly HO and mobility performance, the research community must be aware of these limitations and consider and explore alternative schemes to handle these issues efficiently. Each KPI is described in more detail in the following subheadings:

**HO rate (HOR):** HO rate (HOR) is the frequency or number of HOs that occur within a given time period, such as per second or per hour. A high HOR indicates that the UE is frequently moving from one cell to another or that the network is unable to maintain a stable connection with the UE. Conversely, a low HOR indicates that the network is providing a stable connection to the UE. In 5G HetNet, the HOR tends to increase due to the smaller cell sizes and high-speed UE scenarios. It is generally preferred to maintain a low HOR to ensure a secure and reliable HO process in 5G HetNet. This can be achieved through proper settings of HCPs (such as HOM and TTT).

(1) $${KPI}_{HOR}=\frac{N_{HO}}{unit}$$

**HOF rate (HOFR):** HOFR measures the percentage of HO attempts that fail and is defined as the “total number of failed HOs (HOFs) divided by the total number of HOs (sum of successful HOs and failed HOs)”. HOFs can occur due to various reasons such as poor network planning, heavy cell load or network congestion, ICI, weak RSS, outdated UE, and poor mobility management. Reducing the HOR can help to address HOFR, but there is a tradeoff between minimizing the HOR and HOFR [88].

(2) $${KPI}_{HOFR}=\frac{N_{HOF}}{N_{HO(total)}}$$

**HO success rate (HOSR):** HOSR indicates the percentage of HOs that are successfully completed, which is defined as “the ratio between the total number of successful HOs and the total number of triggered HOs in a particular time” [88]. The HO process is considered successful if it is completed before the RSS from the serving cell drops below a specified threshold level (minimum acceptable RSS level) [89]. A high HOSR means that the number of failed HOs is minimized, the HO process is smooth, and the system is providing seamless connectivity to the UE, while a low HOSR indicates the opposite.

(3) $${KPI}_{HOSR}=\frac{N_{HO}}{N_{HO(total)}}$$

**HOPP rate (HOPPR):** HO Ping-Pong (HOPP) occurs when a UE frequently connects and disconnects with two different BSs due to rapid fluctuations in signal strength (RSSI) from both stations (serving and target BS) [90]. HOPPR measures the frequency of a UE going back and forth between two cells during the HO process. A high HOPPR indicates that the UE moves frequently between two cells, leading to increased call drop rates, poor user experience, and interruptions in data transfer. A low HOPPR indicates that the UE does not frequently move between two cells and that the HO process is smooth.

(4) $${KPI}_{HOPPR}=\frac{N_{HOPP}}{N_{HO(total)}}$$

**HO interruption time (HOIT):** The duration of time during which the HO process is interrupted and the UE is disconnected from the network is called HOIT. During HOIT, the UE does not transmit or receive any information to/from any BS [91]. High HOIT indicates an issue with the HO process, while low HOIT indicates that the HO process is working well.

**HO execution time (HOET):** HOET, also known as HO latency (HOL), is the amount of time from the initiation of the HO process to the completion of the HO phase [92]. High HOET indicates that the HO process is taking longer than usual, while low HOET indicates that the HO process is completed quickly and the network is capable of providing uninterrupted connectivity to the UE within its coverage area.

**HO energy consumption (HOEC):** The amount of energy that a UE consumes during the HO process is called HOEC. High HOEC leads to a reduction in UE battery life, while low HOEC means the UE is consuming minimum energy during the HO process, which enhances the UE battery life [93].

**Radio-link failure rate (RLFR):** RLF is the loss of connection/link between the UE and the network (BS), and the frequency of RLF is known as RLFR. RLF occurs due to several reasons, such as weak RSS, HO to the wrong cell, poor coverage area, and heavy cell load or traffic congestion. Low RLFR is desirable in 5G HetNet for ensuring seamless connectivity to the UE and reducing HOIT.

(5) $${KPI}_{RLF}=\frac{N_{RLF}}{N_{RL(accepted)}}$$

The performance of HO KPIs, namely HOF and HOPP, under different UE speeds and HCP values is illustrated in Table 5. It is evident from the table that HOPP is not a concern in high-speed scenarios irrespective of the HCP values, while HOF issues exist in both high-speed and low-speed scenarios depending on the HOM and TTT values.

### 4.2. Advantages of Mobility Management in 5G HetNet

Mobility is the maximum speed of a UE in km/h at which a specified QoS can be achieved. Mobility performance is critical for any WCS, especially for URLLC services that require low latency and high reliability [94]. Efficient mobility management is essential to achieve the demands of 5G HetNet and includes enhanced capacity, reduced network congestion, lower latency, and uninterrupted connectivity. Previous studies have shown that the demand for mobile services is increasing with the advent of new mobility paradigms, such as self-driving vehicles, drones, and mobile small cells [95].

There are two types of mobility in WCS: cell-level and beam-level. In cell-level mobility, the UE switches from one cell to another and establishes a new connection with the new cell. In beam-level mobility, UEs move from one beam to another within the same cell coverage, establishing a new data path to the new beam. The 5G HetNet supports both cell-level and beam-level mobility to ensure seamless connectivity and high reliability, while LTE-A (4G) only supports cell-level mobility.

Reducing the HOIT and the HOF rate can improve mobility performance in 5G-NR. In 5G-NR, the deployment of UD-HetNet reduces the size of the HO region, which consequently increases the HOF rate due to FHO. The conditional handover (CHO) technique can improve mobility performance, where the UE receives the HO command before the RLF from the S-gNB but does not execute the HO immediately. The DC-based HO, where UE connects with both S-gNB and T-gNB, also reduces HOFs, but it involves high complexity and UE cost [96].

HOIT is the duration during which a UE neither transmits nor receives any data. In 4G/LTE-A, the HOIT is about 10ms, which results in a significant transmission delay. However, 5G-NR technology has reduced this delay to 1ms by utilizing soft handover and RACH-less handover techniques. If the HOIT is large, an HO mechanism that filters out the effect of shadowing and does not attempt a handover is preferable. However, if the HOIT is small, an HO to the best cell, exploiting the shadowing effect, can be beneficial for both cell association and system throughput.

### 4.3. Advantages of HO in 5G HetNets

The HO process in 5G-NR is similar to that in 4G/LTE-A, but 5G-NR outperforms 4G/LTE-A in terms of speed, reliability, and efficiency. The use of beamforming technology in 5G-NR enhances the accuracy of signal measurements, resulting in faster HO decisions. In contrast, 5G’s edge computing technology efficiently reduces HO latency compared to LTE-A. Furthermore, the use of DC technology in 5G-NR improves HO reliability more than in previous generations (4G LTE). DC enables the UE to maintain a connection with the source and target gNBs during the HO process, reducing data packet loss and improving reliability. In addition, the implementation of SDN and NFV in 5G-NR allows the network to be easily reconfigured to meet challenging requirements.

### 4.4. Challenges in Mobility Management for 5G HetNet

The smaller size of cells in 5G HetNet may increase unnecessary mobility requests, resulting in an excessive number of HOs. Despite the numerous advantages of mobility management in 5G HetNet, significant challenges can degrade the system’s performance. These challenges include managing multi-RATs (mmWave and Sub-6GHz), load balancing, FHO, signaling overhead, power and energy consumption, low latency and high reliability, security, scalability, network optimization, and location management. The reasons, consequences, and significant challenges of HO and mobility management in 5G HetNet are further discussed in Section 6.

### 4.5. 5G HetNet HO Issues

**Figure 8:**
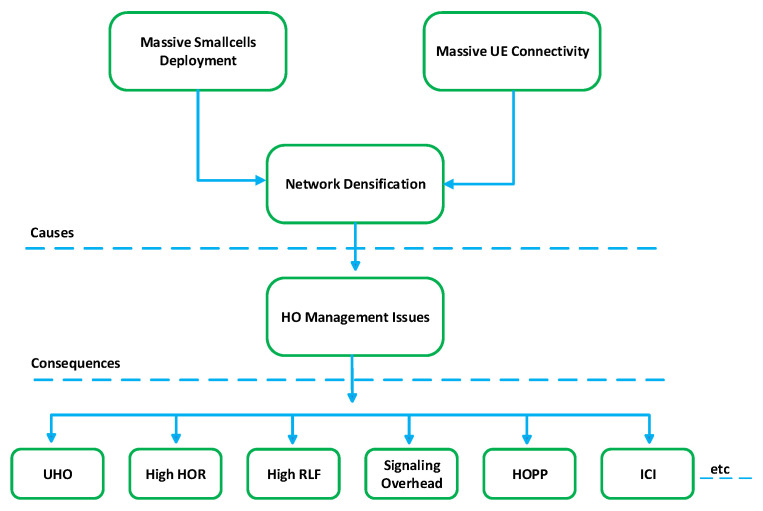
HO issues in 5G HetNet.

The deployment of UDSC and the massive number of connected UEs in 5G lead to an ultra-dense network (UDN), causing several HO management issues. These issues include high HOR, frequent and UHO, HOPP, RLF, signaling overhead, and ICI. These problems can degrade UEs’ HO performance and reduce seamless connectivity in 5G HetNet. Figure 8 illustrates the causes and consequences of HO issues in 5G HetNet.

## 5. Current Solutions for HO and Mobility Management Challenges in 5G HetNet

Effective management of mobility and HO is critical for ensuring high-quality service in NG-WCS. In particular, mobility management poses significant challenges in UD-HetNets in 5G architecture, requiring innovative and enhanced techniques to guarantee secure and uninterrupted mobility. Extensive research has been conducted on mobility management. This article aims to investigate the HO and mobility challenges in 5G HetNet in terms of current mechanisms and propose possible solutions.

In previous sections, we provided a detailed background of 5G technology, HO, and mobility management. This section focuses on state-of-the-art techniques used to address HO and mobility management issues in 5G HetNet. While 5G HetNet offers high performance and throughput, HOF and mobility management are key challenges that must be addressed to ensure seamless connectivity among users.

Various approaches have been proposed by researchers to address HO and mobility management in 5G HetNet. For instance, in [97,98,99], the authors investigate techniques to mitigate HOFs. HO and HOF rates are mathematically evaluated in [100,101,102,103,104]. The performance of HHO in 5G HetNet is analyzed in terms of outage probability (OP) and HO rate in [105]. The analysis is conducted by estimating UE speed against OP and the total number of HOs. In [106], the authors analyze HO performance for HetNet by considering vehicular user velocity, mobility management parameters, and cell size. They investigate HOFs caused by fading and shadowing, and concentric circles are used to model HO locations at picocells. A balanced 5G model is proposed in [107] to organize traffic during the HO process. The mid, high, and low frequencies are selected to investigate the proposed model in terms of network load, active network slice, and type of services. An HO decision algorithm is proposed in [108] using ML techniques to monitor active time, available radio resources, UE speed, and received power of the reference signal. A simulation model is designed based on small and macro cells, carrier frequency (2 to 2.8 GHz), radius, and shared bandwidth. An SDN-based 5G HetNet model is discussed in [109] to address HO authentication issues, and an algorithm called ’dual constraints chaotic radial movement’ is considered for this purpose. An SDN-based 5G HetNet is introduced in [110] to ensure a seamless HO process, and elliptic curve integrated encryption is applied for system privacy and detection of illegal access. A seamless and secure HO process is the focus of [111] with mutual, quick, and strong authentication. The authors developed an SDN model for this purpose, and the paper mainly investigates VHO. Yang et al. [112] presented a 5G SDN-based HetNet using a wireless-link signature scheme to achieve a fast and unified HO process. The model can support macro, small, and femtocells, WiFi, WiMax, and visible light communication. In [113], the authors investigated the increase in interruption time due to HOF and presented a technique called ’zero HOF’ with unforced and automatic time. The HOF is minimized with the enhancement of the HOPP rate. The presented model is discussed using a theoretical model and validated with a simulation structure. The work in [114] utilizes received signal power and speed for adapting TTT and HOM, targets ping pong HOs, and attempts to reduce the standard deviation of balanced load. The proposed system is developed based on performance indicators, control parameters, and system matrices.

Mobility management is a critical and challenging issue for NG-HetNet. In [115], researchers investigated key mobility management issues such as HOPP, HOF, delays, and HOR using radio resources to enhance the performance of 5G HetNet. To improve resource efficiency and QoS, an advanced mobility management and utilization framework is presented in [116], transforming mobility management from a reactive to a proactive state. The mobility prediction model is designed to predict traffic patterns and various attributes of user mobility, such as future cell load, time, and the next candidate cell for HOs. A novel approach based on intuitionistic fuzzy sets is proposed for the QoS estimation of service compositions, which is appropriate for all kinds of networks and is particularly advantageous in the 5G and B5G network paradigm [117].

To achieve the promising QoE and empower the mobility management paradigms for future HetNets, the OPERA framework is proposed in [118]. This technique predicts the next cell load and optimizes CIOs and antenna parameters. The presented technique also investigated coverage, capacity constraints, and load-aware strategies to ensure seamless operations.

Additionally, other latest ML-, D-HCP-, FLC-, and SDN-based schemes for improving HO and mobility performance in 4G/5G cellular systems are listed in Table 6, Table 7, Table 8, and Table 9, respectively. The tables highlight the various HODPs and KPIs achieved and the limitations of these techniques in achieving excellent HO performance in high-speed scenarios.

## 6. Focused Challenges in 5G HetNet

This section discusses various challenges faced by 5G HetNet. Each review article mentioned in the related survey comparison table (Table 2) focuses on specific issues faced by UE during the HO process. This paper discusses problems that degrade the HO performance of 5G HetNet, such as ultra-high speed, load balancing, signaling overhead, ICI, and security and privacy.

The high-speed challenge in 5G HetNet refers to the difficulty of maintaining stable and smooth communication for fast-moving users, such as trains, vehicles, or aircraft. The issue arises because high-speed movement creates a rapidly changing radio environment and frequent and UHO, which can lead to signal interference and fading. These UHOs increase the probability of HOF and RLF, which consequently degrade the network performance. To address this challenge, 5G HetNet uses advanced techniques such as beamforming, massive MIMO, and predictive algorithms to improve signal strength, reduce interference, and optimize the HO process. These technologies work together to provide users with a seamless and reliable network experience, even in high-speed scenarios, to ensure that they can stay connected while moving.

Load balancing is another critical issue in 5G HetNet that arises due to the exponential increase in the number of devices and data volume. This issue arises because network traffic is not evenly distributed throughout, and some BSs may become overloaded while others remain underutilized. This can result in reduced network efficiency, increased latency, and dropped connections. The 5G HetNet uses advanced load-balancing techniques to address this challenge by dynamically distributing network traffic across BSs based on network conditions and resource availability. This ensures optimal network resource utilization, prevents any single BS’s overload, and provides users with a seamless and reliable network experience.

Signaling overhead in 5G HetNet is another significant issue that can impact network efficiency and performance. This issue arises because the HO process between BSs involves multiple signaling messages between the device and the network, which can result in excessive signaling overhead. This can lead to increased HO latency, reduced network efficiency, and dropped connections. To address this challenge, 5G HetNet uses advanced signaling mechanisms that minimize the number of signaling messages required and reduce the burden on the network. This includes using intelligent HO mechanisms, such as predictive and proactive HO, that can anticipate the device’s movement and optimize the HO process. These advanced signaling mechanisms ensure a seamless HO process, improve network efficiency, and provide users with a reliable network experience.

ICI in 5G HetNet refers to signal interference caused by signals from adjacent cells. The challenge arises because the radio frequencies used by different cells overlap, resulting in interference that can degrade signal quality and impact network performance. The ICI issue can lead to dropped connections, reduced network efficiency, and increased latency. To address this challenge, 5G HetNet uses advanced signal processing techniques such as interference coordination and suppression, which can reduce the impact of ICI in 5G HetNet.

Finally, ensuring security and privacy protection for user data from unauthorized access is another critical challenge in 5G HetNet. This issue arises because 5G HetNet supports a wide range of applications, devices, and technologies that require varying levels of security and privacy protection. To tackle this issue while transmitting large volumes of data across 5G networks, 5G HetNet utilizes several advanced end-to-end encryption techniques, user authentication protocols, advanced technologies, and the use of secure virtual private networks (VPNs) to protect user data, ensure network security, and enhance network reliability. These measures ensure that user privacy is protected and 5G networks can be trusted to handle massive data securely.

The main causes and consequences of these challenges are summarized in Table 10.

## 7. Future Directions

In the previous sections, we discussed the background of HO and mobility management for 5G HetNets as well as the existing approaches to address these issues. However, the key challenges for HO and mobility management in future 5G HetNets were not fully explored. In this section, we recommend future directions to ensure the sustained performance of 5G HetNets in terms of HO and mobility management.

Mobility is a key characteristic of users in cellular networks, and it can cause various problems such as HOF, early and delayed beam switching, and inter-system ping pong. Therefore, mobility management is a critical factor behind the applications of wireless cellular networks and their widespread adoption. Furthermore, according to the Ericsson Mobility Report [143], mobile data traffic is expected to increase 287 times between 2017 and 2027, which will exacerbate HO and mobility management issues.

In 5G and B5G HetNet, QoS directly influences the QoE such that poor QoS can result in a lower QoE for the user and vice versa. Additionally, combining multiple services with varying QoS requirements can lead to the issue of QoS composition. If the QoS requirements are not managed and balanced accurately, it may lead to a reduction in QoE for certain services. Therefore, it is crucial to carefully consider and optimize both QoS and QoE in 5G/B5G HetNet to deliver an adequate user experience and enhance HO and mobility performance. The current HO and mobility management models are inadequate in delivering the expected QoS, resource efficiency, and QoE. In this survey paper, we recommend the following strategies to mitigate the HO and mobility issues in 5G and B5G HetNets and enhance the ability to support UE seamlessly.

### 7.1. Kalman Filter

The Kalman Filter (KF) is a recursive mathematical process based on a set of equations that is widely used in many engineering and scientific fields. It is known for its fast convergence speed, efficient tracking, and compensation ability. Using KF-based algorithms for HO and mobility management in 5G HetNets is an important research direction with significant potential benefits.

- Firstly, KF can provide an accurate and real-time estimation of user location and velocity, which is critical for efficient HO and network optimization. This means that the filter can predict the optimal time and location for the next HO and adjust network parameters accordingly to ensure that users stay connected to the network and experience minimal disruption.
- Secondly, the KF can track user movements and predict their future trajectory, allowing for more efficient use of network resources. The filter can reduce interference and improve overall network reliability by adjusting network parameters such as antenna beamforming and transmission power. This can result in better overall network performance and improved user experience.
- Finally, the KF is a flexible and adaptable approach that can be customized to different network scenarios and configurations. This means that it can be used in a wide range of 5G HetNets, including those with different BS volumes, user densities, and network topologies.

### 7.2. Unmanned Aerial Vehicles (UAVs)

Unmanned Aerial Vehicles (UAVs), or drones, offer a promising new solution for improving HO and mobility performance in 5G and B5G HetNets. With UAVs acting as mobile BSs, network coverage and capacity can be extended to remote or disaster-stricken areas that are difficult to reach with traditional terrestrial BSs. UAVs provide several benefits, including improved QoS, reduced call drop rates, and HOF, by serving as mobile BSs for devices with poor signal quality. They can also provide connectivity in areas without ground-based BSs, offload traffic to reduce congestion and improve overall network performance, and serve as essential communication links in emergency situations where ground-based networks are disrupted. Moreover, UAVs can be deployed on-demand in situations where existing BSs are unable to provide optimal service, reducing costs and improving connectivity. In summary, UAV-based solutions have the potential to revolutionize HO and mobility management in 5G and B5G HetNets.

### 7.3. Multi-Connectivity

Multi-connectivity, which allows UEs to connect with multiple BSs simultaneously, can be leveraged to enhance HO and mobility performance in 5G and B5G HetNets. By maintaining multiple connections simultaneously, multi-connectivity can enable seamless HO between different network types, such as 5G, WiFi, and cellular networks. This approach improves network capacity, reduces congestion and network failures, and provides better connectivity and user experience even if one of the connections is interrupted. Multi-connectivity can also improve coverage and signal quality by leveraging multiple networks, provide load balancing to distribute traffic over multiple connections, reduce delays caused by traffic overload, and offer redundancy by maintaining backup connections in case of network failures. Additionally, multi-connectivity enables QoS optimization to select the best network for specific applications based on QoS requirements. By doing so, this approach enhances network efficiency, reduces delays, and ultimately improves HO and mobility performance in 5G and B5G HetNets.

### 7.4. Direction-of-Arrival (DoA) Estimation

Direction-of-arrival (DoA) estimation is critical to improving HO and mobility performance in 5G and B5G HetNets. Accurate DoA estimation helps identify the best serving cell for mobile devices, ensuring a smooth HO process, reducing HOF, and minimizing interference. There are several methods to improve DoA estimation, including massive MIMO, adaptive beamforming techniques, and ML techniques.

- Multiple antennas (massive MIMO) can improve the received signal power and reduce noise and interference.
- Advanced signal processing techniques such as adaptive beamforming can enhance the accuracy of DoA estimation, reduce interference from other directions, and provide a more reliable communication link.
- ML techniques can be used to analyze large datasets of signal measurements, improving the accuracy of DoA estimation.

By implementing these schemes, DoA estimation can be made more efficient, reducing congestion and improving load balancing and leading to better network performance in terms of HO and mobility.

### 7.5. Cooperative Handover

Cooperative HO is a technique that has gained significant attention from researchers and industry experts for enhancing HO and mobility performance in 5G and B5G HetNets. Much research has been done to enhance the mobility and HO performance in 5G HetNets. Cooperative HO can provide several benefits, including reduced HO delay, improved reliability, and enhanced network performance. By sharing information about network conditions and traffic load, source and target BSs can make informed decisions about the HO process, leading to a seamless and efficient HO. Additionally, by exchanging information about neighboring cells and their interference patterns, the source and target BSs can select an HO target with minimum interference, improving the reliability and performance of the network. Overall, cooperative HO can enhance HO performance in 5G and B5G HetNets by facilitating a smooth and efficient HO process through sharing of information and collaboration between the source and target BSs.

### 7.6. Channel Modeling

Channel parameters are one of the major concerns that may influence the performance of the 5G WCS and include HO decisions and mobility management protocols. Therefore, to analyze, build, and optimize mobility management in 5G HetNets, adequate channel modeling plays a key role. Accurate channel modeling enables efficient allocation of radio resources, intelligent beamforming, and antenna selection, ensuring seamless HOs and an improved user experience. We believe that stochastically and deterministically based wireless channel modeling can optimize mobile data traffic between the sender and receiver, especially in high-speed moving objects. Path loss, signal fading, and multiple component parameters can be estimated and minimized through channel modeling, thus achieving seamless connectivity among mobile users.

## 8. Conclusions

Effective mobility management is crucial for ensuring the expected QoE and QoS in 5G HetNet. The HO process plays a critical role in mobility management, and failures during this process can have adverse effects on the reliability, stability, and connectivity of 5G communication networks. This survey paper reviewed various techniques to address mobility and HO challenges in 5G HetNet. We provided a detailed background of HO and mobility and explained how QoS, connectivity, and data rates are affected in 5G UD-HetNets. We also discussed the architecture of 5G and various 5G technologies, including HetNet, mm-Wave, massive MIMO, full duplex, etc. We further investigated the HO process and mobility management in 5G UD-HetNet, covering various events, conditions, decisions, control parameters, and KPIs. After discussing the HO and mobility management in 5G UD-HetNet, we compared recent structures that mitigate HO and mobility management issues based on KPIs, HODPs, and HCPs while highlighting their limitations. Finally, we examined the future-focused challenges in 5G UD-HetNet associated with the HO process and mobility management, including security and privacy, ICI, load balancing, signaling overhead, and high speed. In the last section, we provided possible efficient and optimized future directions and recommendations. By addressing the challenges discussed in this paper and implementing future directions and recommendations, we can ensure a seamless and reliable 5G HetNet with improved QoE and QoS, providing users with an enhanced communication experience.

## Figures and Tables

**Table 1:** Comparison of related surveys on HO and mobility management with our survey paper.

Ref.	Description	Considered Platform
[28]	This comprehensive review examined HO techniques over DC and innovative solutions such as ML-based approaches in future UD-HetNet to address the mobility management issues in LTE-A and advanced WCS.	LTE-A/5G UD-HetNet
[29]	The mobility management general concept, possible research directions, and major challenges such as HO issues, signaling overhead, power consumption, security, and latency faced by upcoming new WCS were discussed in detail. Moreover, some effective solutions were demonstrated to meet/encounter future network mobility management requirements.	5G HetNet
[30]	Based on 5G architecture and service types, a brief survey was conducted in this paper to explore the concept of VHO in the future wireless system. In addition, it also analyzed how SDN/NFV and MEC technologies can be utilized to manage mobility and HO issues in NG-WCS.	5G-HetNet
[31]	It examined previous research on mobility and HO management, characterizations, and fundamental causes of mobility issues in UD-HetNet. It provides the fundamental causes of mobility challenges in future networks and highlights potential solutions and opportunities for upcoming development. An overview of the challenges and practical considerations to be addressed in NG networks is also provided.	5G UD-HetNet.
[32]	This paper discussed the key enabler technologies required to improve the QoS and meet the NG-WCS requirements. NG-WCS background, benefits, and different network layouts were studied extensively. Moreover, existing ML-based techniques to overcome the challenges related to mobility and HO in NG-WCS are also reviewed in detail.	5G
[33]	A comprehensive review and analysis of various ML-based techniques for HO management in future dense network environments are carried out in this article. Several open challenges with possible solutions based on ML are also suggested to improve the future network performance	5G UD-HetNet
[34]	In this paper, the HO management challenges and HO decision algorithms for LTE-A are studied in detail. The pros and cons of each HO decision scheme based on input system parameters and KPIs are also reviewed.	LTE-A/4G/HetNet
[35]	This article extensively reviewed various HO self-optimization schemes, particularly MRO and LBO, for evaluating the seamless HO in NG-WCS. This article also highlighted the pros and cons of the previously proposed schemes, challenges faced by HO self-optimization schemes, and possible solutions to overcome the mobility and HO issues in future HetNet environment.	5G
[36]	The basic HO execution procedure and HO management overview in LTE-A and NR are discussed. Moreover, the general concept of radio access mobility (RAM), such as idle and connected mode mobility and possible challenges related to RAM in cellular networks, are presented.	5G-NR
[37]	The authors address the 5G-enabling key technologies, challenges, and opportunities related to ML-based HO management schemes and possible solutions to address these challenges.	5G UD-HetNet
This Study	In this study, we explain the general concept of 4G and 5G. The architecture, services, and technologies of the 5G system to improve mobility and HO issues in future WCS are also discussed. Moreover, various schemes such as SDN, ML, FLC, and dynamic HO control parameters (D-HCPs) are also examined to improve the HO performance in 5G HetNet. Finally, important future challenges that degrade the HO and mobility performance in 5G systems and possible recommendations for solutions are also discussed in detail.	5G UD-HetNet

**Table 2:** Related surveys, discussed scenarios, schemes, and focused challenges.

Ref.	Scenario				HO/MM	Schemes				Focused Challenges				
	4G	5G				ML	FLC	SDN	D-HCP	High-Speed	LB	ICI	Overhrad	Security
		Services	Architecture	Technologies										
[28]	✓		✓	✓	✓	✓		✓	✓	✓				✓
[29]		✓	✓		✓	✓	✓	✓	✓		✓		✓	✓
[30]		✓	✓		✓			✓	✓					
[31]		✓			✓	✓			✓		✓		✓	
[32]		✓	✓	✓	✓	✓		✓				✓		✓
[33]		✓	✓	✓	✓	✓	✓				✓	✓	✓	✓
[34]	✓			✓	✓	✓		✓			✓	✓		
[35]	✓	✓	✓	✓	✓	✓	✓			✓				
[36]	✓		✓		✓	✓	✓		✓	✓				
[37]		✓	✓	✓	✓	✓					✓	✓	✓	✓
**This Work**	✓	✓	✓	✓	✓	✓	✓	✓	✓	✓	✓	✓	✓	✓

**Table 3:** Various 5G key technologies and achievements.

5G Technologies	Achievements	Description
M-MIMIO + UD-HetNet	Improved capacity, data rate, and coverage	Uplink data rate = 10 Gbps
mm-Wave + UD-HetNet	Increased SE and connectivity, reduced latency	SE > 20 b/s/Hz, 10^6^ users/km^2^
Beamforming + UD-HetNet	Enhanced reliability and QoE, mitigates interference	100% availability (signal quality) and 99.99% reliability
M-MIMIO + UD-HetNet + mm-Wave	Reduced energy consumption and enhanced coverage in UD and high-mobility scenarios	Supports up to 500 km/h user mobility
SDN/NFV + UD-HetNet	Improved QoS, security, and scalability	Improved system security and scalability
FD communication + UD-HetNet + D2D communication	Minimizes the traffic load and enhances network capacity (SE)	Massive connectivity of UEs

**Table 4:** Various HO events and entry regulations.

S. No	Event Occurrence	Entering Condition/Rule
1	A_1_	When the S-gNB RSS exceeds the threshold level.
2	A_2_	When the S-gNB RSS drops below the threshold level.
3	A_3_	When the T-gNB RSS is stronger than that of S-gNB.
4	A_4_	When the RSS from the T-gNB exceeds the threshold level.
5	A_5_	When the RSS from the S-gNB drops below Threshold 1 and the T-gNB RSS exceeds Threshold 2.

**Table 5:** HO KPI performance for various UE speeds and HCP settings.

Speed Scenario	TTT	HOM	HOPP	HOF
High	Minimum	Minimum	Low	Low
High	Maximum	Minimum	Low	High
High	Maximum	Maximum	Low	High
High	Minimum	Maximum	Low	High
Low	Minimum	Minimum	High	High
Low	Maximum	Minimum	Low	High
Low	Minimum	Maximum	High	Low
Low	Maximum	Maximum	Low	Low

**Table 6:** Summary of latest ML-based schemes for improving HO performance in 4G/5G HetNet.

Ref.	Technology	HODPs	KPIs	Applied Mechanism
[108]	5G HetNet	Speed and RSRP	HOPP, HOR, and QoS	ML
[119]	LTE-A	Speed, cell load, and HO history	HOF, pp. rate, and throughput	RL
[120]	5G	Speed, cell load, and RSS	HOP and HOSR enhancement	NN
[121]	mm-Wave HetNet	Speed, RSS, and cell radius	HOF, RLF, HOIT, and EE	KNN-ML
[122]	5G HetNet	User mobility	HOR and throughput enhancement	DRL and DNN
[123]	Muli-RAT HetNet	Speed, RSS, and power	HOR, EE, and QoS	RL
[124]	5G HetNet	Speed, SINR, and PLR	HOSR	NN
[125]	5G UD-HetNet	SINR, cell load, and mobility	HOR, HOFR, RLF, and throughput	Q-Learning

**Table 7:** D-HCP (TTT and HOM)-based schemes for improving HO performance in 4G/5G HetNet.

Ref.	Technology	HODPs	KPIs	Maximum
				Considered Speed
[2]	HetNet	Speed and RSRP	HOF, CDR, IT, and HO delay	160 km/h
[126]	HetNet	Speed and RSRP	HOR, HOF, and HOPP	130 km/h
[127]	LTE-A and 5G HetNet	Speed	RLF, HOPP, and SE	160 km/h
[128]	5G	Speed and RSRP	HOPP, RLF, and HOP	200 km/h
[129]	5G	Speed and cell load	HPPP, OP, and HOP	140 km/h
[130]	4G and 5G HetNet	Speed and RSS	HOPP and RLF	160 km/h
[131]	4G and 5G HetNet	Speed	HOPP, RLF, and HOIT	160 km/h

**Table 8:** FLC schemes based on various HCP values for improving HO performance in 4G and 5G HetNet.

Ref.	Technology	HODPs	KPIs	Comments
[132]	4G/5G HetNet	Speed, SINR, and cell load	HOPP, RLF, and HO latency	Cell sectorization approach is utilized; CIO can be used to further enhance HO performance
[133]	Dense HetNet	Speed and channel quality	HOR, HOF, and HOPP	HO KPIs are evaluated on the basis of the number of SCs deployed.
[134]	LTE	Speed	HOR, HOPP and HO delay	The proposed FLC scheme only enhances HO performance in LTE rather than 5G. Target cell load has not been considered for HO
[135]	5G HetNet	Speed, cell load, and RSRP	HO delay, throughput, and coverage	The article does not consider practical implementation challenges of mm-Wave small cells in HetNet
[136]	LTE-A and 5G HetNet	Cell load, RSRP, and SINR	Load level, CDR, and throughput	The proposed algorithm can be applied to various types of HetNet, including mobile networks and cloud computing environments

**Table 9:** SDN-based schemes for improving HO performance in 4G and 5G HetNet.

Ref.	Technology	HODPs	KPIs	Drawbacks/Recommendation
[137]	5G UD-HetNet	Speed and network densification	HOF and HO delay	User speed up to 100 Km/h was taken into account; the effects of HOPP, FHO, and UHO on HO delay was not investigated
[138]	5G UD-HetNet	Speed, location, and direction	HOF and HO delay	Only number of UE variation is considered to measure the HO KPIs; SDN can be utilized for radio resource allocation and management in 5G UD-HetNet
[139]	5G UD-HetNet	RSRP, SINR, and cell load	HOF and HOPP	Difficult to obtain the optimal HO trigger point in the case of drastic changes in the network
[140]	5G HetNet	Speed, RSS, and application type	HOR, signaling overhead, HO delay, and throughput	Enhances the mobility management of the user within 5G Femto-eNodeB HetNet only
[141]	5G HetNet	RSSI, SNR, and RAN load	HOR, latency, and throughput	Vehicle speed of 36 km/h taken into account; inadequate solutions for SDN-based mobility and HO management in MEC-enabled vehicular networks
[142]	5G	Cell load and RSSI	HO delay and throughput	Homogeneous network assumption limits proposed scheme’s applicability to HetNet vehicular environments

**Table 10:** Description of focused challenges in 5G HetNet.

Challenge	Reason	Consequences
High Speed	It occurs due to limitations in equipment capabilities, insufficient network coverage, interference, signal attenuation, and insufficient backhaul capacity.	Increases the number of UHOs; reduces network capacity and network performance in terms of HOF and RLF rate.
Load Balancing	Due to irregular user distribution, cell size, capacity differences, ineffective HO mechanisms, and limited coordination between network layers.	Heavily loaded cells due to load imbalance can cause high HOF, uneven utilization of resources, and degraded QoS.
Signaling Overhead	A large number of small cells and frequent HOs due to their small coverage area is the main cause of increased signaling overhead.	The higher the HOR, the higher the signaling overhead will be, which consequently increases the HOIT and HO delay in WCS.
ICI	It occurs due to high user density, overlapping cell coverage, and inadequate frequency reuse.	Increased call drop rate (CDR) and degraded user satisfaction due to overlapping cell coverage area.
Security and Privacy	Network densification (huge number of cells and connected UE) and multiple technologies and interfaces.	Increased risk of cyber attacks, compromised user data, and reduced trust in network reliability and integrity.

## Data Availability

All the data are available in the paper.

## Abbreviations

The following abbreviations are used in this manuscript:

3GPP	3rd-Generation Partnership Project
5G	Fifth Generation
5GC	5G Core
B5G	Beyond 5G
BBM	Break-Before-Make
BS	Base station
CHO	Conditional Handover
CIO	Cell Individual Offset
CSI	Channel State Information
D2D	Device-to-Device
DoA	Directional of Arrival
DRL	Deep Reinforcement Learning
eMBB	Enhanced Mobile Broadband
EPC	Evolved Packet Core
FD	Full Duplex
FHO	Frequent Handover
FLC	Fuzzy-Logic Controller
HCP	Handover Control Parameter
HD	High-Definition
HetNet	Heterogeneous Network
H-HO	Hard Handover
HHO	Horizontal Handover
HO	Handover
HPPP	Handover Ping-Pong Probability
HODP	Handover Decision Parameter
HOET	Handover Execution Time
HOF	Handover Failure
HOFR	Handover Failure Rate
HOIT	Handover Interruption Time
HOM	Handover Margin
HOPP	Handover Ping-Pong
HOSR	Handover Success Rate
ICI	Inter-Cell Interference
ICIC	Inter-Cell Interference Coordination
IoT	Internet of Things
ITU	International Telecommunication Union
KF	Kalman Filter
KNN	K-Nearest Neighbors
HOPP	Handover Ping-Pong
HOSR	Handover Success Rate
ICI	Inter-Cell Interference
ICIC	Inter-Cell Interference Coordination
IoT	Internet of Things
ITU	International Telecommunication Union
KF	Kalman Filter
KNN	K-Nearest Neighbors
KPI	Key Performance Indicator
LB	Load Balancing
LBO	Load-Balancing Optimization
LTE	Long-Term Evolution
MBB	Make-Before-Break
MBS	Macro Base station
MC	Macro Cell
ML	Machine Learning
M-MIMO	Massive Multiple-input–Multiple-output
mMTC	Massive Machine-Type Communication
mm-Wave	Millimeter Wave
MR	Measurement Report
MRO	Mobility Robustness Optimization
NFV	Network Function Virtualization
NG	Next-Generation
NN	Neural Network
NR	New Radio
NSA	Non-Standalone
OP	Outage Probability
QoE	Quality of Experience
QoS	Quality of Service
RAT	Radio Access Technology
RL	Reinforcement Learning
RLF	Radio Link Failure
RLFR	Radio Link Failure Rate
RSRP	Reference Signal Received Power
RSRQ	Reference Signal Received Quality
RSS	Received Signal Strength
RSSI	Received Signal Strength Indicators
SA	Standalone
SC	Small Cell
SDN	Software-Defined Networking
SE	Spectral Efficiency
S-gNB	Source gNB
S-HO	Soft Handover
SNR	Signal-to-Noise Ratio
TEHO	Too-Early Handover
T-gNB	Target gNB
Time-to-Trigger	TTT
TLHO	Too-Late Handover
UAV	Unmanned Aerial Vehicle
UD-HetNet	Ultra-Dense HetNet
UDSC	Ultra-Dense Small Cell
UE	User Equipment
UHO	Unnecessary Handover
URLLC	Ultra-Reliable Low-Latency Communication
V2V	Vehicle-to-Vehicle
V2X	ehicle-to-Everything
VHO	Vertical HO
WCS	Wireless Communication Systems
